# A knowledge translation toolkit for maternal health implementation planning in low- and middle-income countries: development and pilot evaluation in two countries

**DOI:** 10.1136/bmjgh-2024-018616

**Published:** 2025-11-29

**Authors:** Lisa M Puchalski Ritchie, Kathleen Gamble, Mercedes Vila Ortiz, Edgardo Abalos, Mary Ani-Amponsah, Mariana Duhau, Christine Fahim, Sharon E Straus, Azmach Hadush, Fernando Althabe

**Affiliations:** 1 Faculty of Medicine, Department of Medicine, University of Toronto, Toronto, Ontario, Canada; 2 knowledge translation program, St Michael’s Hospital Li Ka Shing Knowledge Institute, Toronto, Ontario, Canada; 3 Emergency Medicine, University Health Network, Toronto, Ontario, Canada; 4 Institute of Health Policy, Management, and Evaluation, University of Toronto, Toronto, Ontario, Canada; 5 CREP, Rosario, Santa Fe Province, Argentina; 6 Department of Global Public Helath, Karolinska Institute, Stockholm, Stockholm County, Sweden; 7 Maternidad Martin, Mariano Moreno, Rosario, Argentina; 8 School of Nursing and MIdwifery, University of Ghana, Accra, Greater Accra Region, Ghana; 9 National Directorate of Quality in Health Services and Health Regulation, Ministry of Health of the Nation Argentina, buenos aires, Argentina; 10 National Directorate of Maternity, Childhood, and Adolescence, Ministry of health of the nation Argentina, buenos aires, Argentina; 11 WHO Country Office for Zambia, World Health Organization, Lusaka, Lusaka province, Zambia; 12 Institute for Clinical Effectiveness and Health Policy, Buenos Aires, Buenos Aires, Argentina

**Keywords:** Maternal health, Global Health

## Abstract

**Background:**

Knowledge translation (KT) approaches have been advocated to increase uptake of evidence to improve maternal health outcomes in low- and middle-income countries (LMICs). However, their use is limited by lack of KT capacity and limited applicability of many existing KT tools to the unique challenges of LMIC health settings. We developed and evaluated a toolkit designed for use by non-experts and tailored to support implementation planning in LMICs.

**Method:**

Based on our prior research which identified common implementation barriers across five LMICs, a literature review and a qualitative study with women and families in two LMICs, we developed a preliminary item list. Through consultation with our international partners, the item list was refined, a draft toolkit developed and usability tested.

Pilot evaluation of the toolkit employed observation and focus groups with participants at implementation planning meetings conducted in Argentina and Ghana, focused on locally identified evidence-based maternal health implementation priorities.

**Results:**

31 interested parties participated, 10 in Argentina and 21 in Ghana, representing a range of roles relevant to implementation in the local contexts including providers, health educators, policy/decision makers, researchers and patients/patient representatives. Participants reported a number of benefits to the content and organisation of both the toolkit and meeting format, which they noted encouraged open exchange of perspectives and experiences, and comprehensive consideration and discussion of barriers and facilitators (BFs) to implementation in their context. Minor changes to the instructions and wording of a few BFs were suggested and incorporated.

**Conclusion:**

The toolkit provides a resource to support LMIC maternal health implementers by offering a structured approach to assessment and ranking of BFs to implementation and a guide to mapping BFs to evidence-based implementation strategies. Further evaluation across a wider range of health topics and LMICs and in low-resource contexts in high-income countries is needed.

**Study registration:**

*Open Science Framework* https://osf.io/328dy Registered 31 May 2023.

**WHAT IS ALREADY KNOWN ON THIS TOPIC**

- Maternal mortality rates remain unacceptably high, with the vast majority of maternal deaths occurring in low- and middle-income (LMICs) as a result of causes for which effective interventions exist but are not optimally implemented
- Knowledge translation (KT) approaches have been advocated to improve evidence implementation but lack of KT capacity and limited applicability of many existing KT tools to the unique challenges of LMIC health systems limit their use.

**WHAT THIS STUDY ADDS**

- The maternal health implementation planning toolkit provides an evidence-based structured approach to assessment and ranking of barriers and facilitators (BFs) to implementation of evidence-based guidelines, practices and/or policies and a guide to support mapping of BFs to potential implementation strategies to address identified BFs.
- We found the toolkit to be well received by participants who reported a number of benefits to the content and organisation of both the toolkit and meeting format, with minor changes to the instructions for use and wording of a few BFs listed suggested and incorporated to improve usability.

**HOW THIS STUDY MIGHT AFFECT RESEARCH, PRACTICE OR POLICY**

- While further evaluation of the toolkit is needed, the toolkit holds promise as a resource to support maternal health implementers, particularly in LMIC, efforts to improve uptake of evidence and maternal health outcomes.

## Background

 Despite progress, maternal mortality rates remain unacceptably high with an estimated 800 lives lost worldwide daily as a result of pregnancy or childbirth.^1^ Given this less than desired progress to date, reducing maternal mortality remains a priority in the United Nations sustainable development goals.^2^ 95% of maternal deaths occur in low- and middle-income countries (LMICs) where maternal mortality rates were 430 per 100 000 live births veruss 13/100 000 live births in high-income countries.^1^ Deaths result from causes for which proven interventions exist but are not optimally implemented^3^ such as post-partum haemorrhage, post-partum sepsis and eclampsia.^1^ These deaths occur despite the availability of high-quality guidelines produced and disseminated by the WHO, which were developed to incorporate options addressing the unique challenges facing LMIC implementers, such as alternative medication options for management of post-partum haemorrhage where refrigeration of oxytocin is not possible.

For nearly two decades, knowledge translation (KT) approaches have been advocated to reduce the acknowledged know-do gap in LMICs, with the need to build KT capacity a recognised step toward improving uptake of evidence and patient outcomes.^4^ While progress has been made, more is needed. Our KT experience in various countries and the growing KT literature highlight that implementers worldwide are struggling with how to optimise implementation and use existing evidence to inform these efforts.^3 5^ Evidence from systematic reviews shows that theory is seldom used to select or develop implementation strategies.^6^,^8^ As a result, implementation strategies often fail to address key obstacles to implementation both in general and those specific to the local setting. Instead, traditional approaches such as printed education materials are used, which are minimally effective and likely beneficial only if knowledge is the principal implementation barrier.^9^ In our experience in conducting barriers and facilitator (BF) assessments across a range of clinical areas and maternal health guideline implementation planning workshops in six LMICs to date, this is rarely the case with system barriers such as access to medications necessary for implementation predominating in many LMICs.^10^,^12^

Effective implementation planning involves a complex series of steps that are often resource intensive and dependent on local KT capacity. Both are frequently not available in many high-burden LMICs where improved implementation could achieve the greatest benefit. Once implementation targets are prioritised, implementation planning begins with identification of BFs to implementation. Identified BFs are then mapped to theory-informed, evidence-based implementation strategies adapted to the local political, clinical, health system and cultural context.^3^ Doing so increases the chance that an intervention will be both effectively implemented and successful in improving outcomes.^13^

Prior work conducted by our group identified common and unique BFs impacting implementation activities across a range of LMICs and clinical areas.^11^ Based on this work, our partners identified a need for a toolkit for use by non-experts (individuals without specific training or expertise in KT) to support effective implementation planning to optimise uptake of evidence in general, and WHO maternal health guidelines in particular, with the ultimate goal of improving maternal health outcomes.

The purpose of this article is to describe the development and pilot evaluation of a toolkit to guide assessment of BFs to implementation of maternal health evidence-based clinical guidelines, policies or health system interventions in LMICs and to support mapping of BFs and selection of evidence-based implementation strategies to address identified BFs.

## Method

### Development of draft toolkit

#### Step 1: Item generation and selection

A preliminary BF list was developed based on prior research conducted by our group which identified common and unique BFs to implementation of maternal health knowledge products through secondary analysis of findings of implementation projects in five LMICs.^11^ As our prior work examined patient level BFs predominantly from the provider, academic and policy/decision maker perspective,^11^ the preliminary list was supplemented with data from (1) a review of the literature, including a recent systematic review^14^ of barriers to accessing maternal healthcare in LMICs, and (2) findings of a qualitative study assessing patient level BFs from maternal age women and their families in two LMICs (Argentina and Ethiopia) (unpublished data).

#### Step 2: Item refinement, Organisation and Toolkit format

The preliminary BF list was shared with the study team and additional internationally based partners recruited by email through the study teams’ networks, and the item list refined and organised based on the feedback received. Two items identified in our preliminary work, specific to health conditions unrelated to maternal health, were eliminated. In addition, several items grouped in the preliminary work were deemed sufficiently different to warrant inclusion as distinct BF in the draft toolkit. Items were organised according to the level at which they affect implementation, with patient/community level items presented first, followed by provider level and finally health system level. Based on past experience and in consultation with our international partners, this ordering was selected to ensure adequate consideration of BF at all levels. Similarly, as barriers are often more readily identified, facilitators were included as a distinct category to ensure they are specifically considered.

Given the issues with bandwidth, internet access and prolonged electrical outages, common in many LMIC settings, the toolkit was developed as a website that allows the toolkit to be used online, downloaded or printed for use.

### Patient And public involvement

Women with lived experience and patient representatives participated in the pilot evaluation as meeting participants. A meeting report was provided to both sites and shared by the local teams with participants including patients and patient representatives. Patients and public were not otherwise involved in the design or conduct of the study.

### Usability testing

The draft toolkit was usability tested with international partners recruited by email through our study teams’ networks. Participants were selected who were based in or had extensive experience working in LMIC health settings, in a variety of roles, including policy/decision makers, researchers and healthcare providers, with several participants fulfilling more than one role. Usability testing was conducted by the principal investigator (PI) in person or remotely using video conferencing. Participants were asked to think out loud while working through a hand washing guideline example using the toolkit. The hand washing guideline was chosen for its’ relevance to all healthcare contexts and recognised need for improved implementation globally.^15^

At the end of the think out loud process, participants were asked to rate the toolkit using the System Usability Scale^16^ and probed regarding their experience using the toolkit and any suggestions for improvement. After an initial usability testing, with a usability score of 65, additional details were added to the instructions for use of the toolkit. Following this, three additional rounds of usability testing were conducted, with scores ranging from 77.5 to 92.5, with three consecutive scores ≥70 considered above average.^16 17^

#### Pilot evaluation

Pilot evaluation of the toolkit employed qualitative methods including observation and focus groups with meeting participants. Implementation planning meetings focused on locally identified evidence-based maternal health implementation priorities were conducted in two LMICs, Argentina and Ghana. Argentina and Ghana were selected from our network of international collaborators, as they vary substantially in maternal health outcomes and represent significantly different healthcare systems, and therefore were anticipated to have substantially different BF distributions relevant to implementation of evidence-based maternal healthcare.

In 2020, Argentina’s maternal mortality rate was estimated at 44.9 deaths per 100 000 live births,^18^ 90% of women attended four or more antenatal care visits and 99% of women were attended by a skilled birth attendant.^19^ The maternal mortality rate in Ghana in 2020 was 263.1 per 100 000 live births.^18^ In 2022, 88% of women attended four or more antenatal care visits, and 88% of women were attended by a skilled birth attendant.^20^

Argentina is classified as upper middle income and Ghana as lower middle income.^21^ Argentina is largely urbanised, with more than 92% of the population living in urban areas, compared with 59% living in urban areas in Ghana.^22^ The adult literacy rate in Argentina was 99% in 2018 and 80% in Ghana in 2020.^23^ Although direct costs of maternal healthcare are intended to be paid through government programmes in both countries since the early 2000s, patient access to coverage is not optimal in Ghana due to fees required to join the national programme^24^ and out-of-pocket costs for medications and supplies not covered or not available on site at the time of delivery.^25^ In addition, costs for travel to access care are prohibitive for some patients in both settings.

Implementation topics were selected by the Argentina and Ghana study teams in collaboration with their local networks. The Argentina meeting was focused on improving implementation of prevention and treatment of anaemia in pregnancy as outlined in WHO antenatal care guidelines,^26^ with anaemia in pregnancy recognised as a contributor to preventable maternal mortality. The Ghana meeting was focused on improving implementation of guidelines for prevention and treatment of post-partum haemorrhage, as outlined in the Ghana Ministry of Health Standard Treatment Guidelines.^27^

#### Data collection

Meetings were held in person over 2 days, led by the local study team, with a range of interested parties relevant to the locally identified priority for implementation, including: providers, researchers/academics, policy/decision makers and patients/patient representatives, invited to participate. The Argentina meeting was held on 8 June 2023 and 9 June 2023 and the Ghana meeting, on 17 August 2023 and 18 August 2023. No changes to the toolkit were made between the two meetings.

Day one was led by the local study team in the official language (Spanish in Argentina, and English in Ghana). An introduction to the study, study team and implementation topic, including details of the evidence-based guideline and local data highlighting need for improved implementation, was provided collaboratively by the Toronto PI (LMPR) and local study team leads (EA, MVO and MA). Participants then worked through the toolkit to identify BFs to implementation, through a combination of large and small group discussions. Participants worked independently, guided by the toolkit instructions, with no input from the Toronto study team. Meetings were recorded, and observations conducted by the Toronto-based study team, with inline translation provided by professional interpreters in Argentina. At the conclusion of the toolkit testing, a focus group was conducted with meeting participants, using a semi-structured focus group guide (see online supplemental appendix 1). Focus groups were led by the LMPR in English, with inline translation provided by MVO who is a socio-linguistic Spanish-English translator in Argentina. Focus groups were recorded and transcribed verbatim for analysis.

Day two of the meeting was led by LMPR and began with a review of BFs identified on day one to ensure that participants were satisfied with the list and importance ratings applied to the BFs listed. Once the BF list and rankings were finalised a BFs mapping exercise was conducted to develop a preliminary list of strategies to address identified BFs. Meeting proceedings were again recorded and observations conducted by the Toronto study team.

Written informed consent was obtained from all participants.

#### Analysis

Observation notes and focus group transcripts were analysed independently by two Toronto study team members (LMPR, KG), using qualitative content analysis approach.^28^ KG is an experienced non-clinician healthcare researcher, with no prior experience in either study site. LMPR is an emergency physician and clinical epidemiologist, with extensive experience in implementation in LMIC settings. Nvivo 12 (QSR International Inc., Southport, UK) was used to code and organise data into themes, and to assess for differences/similarities across sites. A draft coding scheme was created based on the data collected through the observation notes and focus group transcripts. The framework was revised collaboratively following an initial round of independent coding, and the revised coding scheme was then applied independently, with no further discrepancies or revisions required.

#### Techniques employed to enhance trustworthiness

Methods utilised during data collection and analysis to enhance the trustworthiness of our study findings included: integrative reflexivity, triangulation across data sources, audit trail and member checking.^29^

## Results

A total of 31 interested parties, 10 in Argentina and 21 in Ghana, participated in the pilot evaluation. Participants represented a range of roles relevant to the implementation of the selected evidence product in the local context and included: providers, health educators, policy/decision makers, researchers and patients/patient representatives. Characteristics of pilot evaluation participants are provided in table 1.

**Table 1:** Participant characteristics

Participant role	Argentina (n=10)*	Ghana (n=21)*
Obstetrician/gynaecologist	3	2
Physician	3	0
Midwife	1	11
Nurse	0	2
Social worker	1	0
Patient/patient representative	1	2
Health educator	0	3
Policy/decision maker	1	1
Researcher	1	1

* Numbers greater than participant total as some participants fulfil more than one role.

### Toolkit content

Participants noted that while not all BFs included in the toolkit would be relevant to all topics in their context, all key BFs were included. The comprehensive BF list was seen as beneficial to facilitate consideration of BFs relevant across the spectrum of maternal health evidence-based implementation topics. While no additional BFs were suggested, participants appreciated the toolkit’s flexibility, with spaces available to add new or unique BFs if needed. A few BFs were noted to lack clarity. Group discussion confirmed their relevance and recommended they be retained with examples added to improve clarity. See table 2, revised items.

**Table 2:** Barriers and facilitators revised to improve clarity

	Original wording	Revised wording
Provider level barriers	Lack of communication/interprofessional collaboration, ethnic/cultural differences, lack of cooperation/blaming	Lack of communication/interprofessional collaboration, ethnic/cultural differences, lack of cooperation/blaming within and across healthcare worker cadres
Health system level facilitators	Inclusion of ‘aspirational’ aspects of guideline: keep for rapid incorporation when able vs concern inclusion now is confusing	Inclusion of ‘aspirational’ aspects of guideline: keep for rapid incorporation when able vs concern inclusion how is confusing (eg, consider whether to include a recommended medication or treatment that is not currently available/approved in your setting in the implementation plan, so that it can be used as soon as available/approved or whether including it before available/approved may cause confusion)
	Legal mandates, regulations, laws, etc	Legal mandates, regulations, laws, etc (eg, advocate for change to address laws or regulations preventing implementation)

The final section of the toolkit, which provides guidance for mapping BFs to evidence-based implementation strategies, was not formally tested. However, participants engaged in a practice mapping exercise in small groups in Ghana and as a large group discussion in Argentina at the end of day two. Although the majority of participants used the mapping chart appropriately, some suggested strategies not listed and of unproven effectiveness.

### Toolkit format

Participants commented that the ordering of BFs within the toolkit was of benefit in guiding discussions. It began with patient/community level BFs, followed by provider level BFs and ended with health system level BFs. They felt that this ordering helped to ensure that BFs at all levels were considered, noting that it was easy to get focused on system issues and not pay sufficient attention to other important areas. Participants also felt this ordering helped them to consider issues that might not be relevant in their local context, but important more broadly for large scale implementation projects, such as BFs unique to rural settings. Participants also generally agreed that ordering BF lists within the levels was optimal, encouraging thorough discussion of issues at each level independently. In addition to the spaces included to allow for addition of BFs not listed, participants felt that the spaces provided beside each BF were helpful for recording specific details regarding the impact of the selected BFs. The details recorded were noted to be a valuable reference later in the process when mapping BFs to implementation strategies.

Internet instability was encountered during the meetings at both sites, highlighting the importance of the option for offline use of the toolkit. In addition to internet instability common in many LMICs, participants noted that electricity challenges can occur in some regions in their settings, necessitating use of a hard copy version of the toolkit. Further, participants appreciated having a print copy of the toolkit in front of them during small and large group discussions, with many making notes to bring forward to the group or to keep for future reference. Participants noted, however, that some clarification for use of the toolkit in print version would be of value, including how to mark and rank their BF selections on the print version. In addition, in preparing the print version for use during the meeting, the study teams found some format spacing changes needed to clearly separate BFs and levels and suggested that including a link to the print version within the toolkit would be beneficial.

### Toolkit instructions

While participants reported that the instructions for use of the toolkit initially provided were generally clear, a number of additions were suggested. Suggestions were similar across the two sites and included additions for all versions, as well as some unique to use of the toolkit in hard copy or computer-based formats.

Suggested additions relevant to all formats included: noting the importance of a group leader and consideration of options to list the topic on the toolkit itself or to post the topic visibly in the meeting space to help discussions stay on track; recommending that sections be read through completely before beginning BF selection as BFs are often not all visible on one screen or page; clarification that not all BFs will apply to all topics, that BF may be actual or perceived, and that only BFs identified as relevant to the specific topic should be selected and ranked. In addition, in observing groups working with the guide to mapping BFs to implementation strategies, the study team noted that additional directions for use of the mapping chart would be beneficial.

Suggested additions specific to use of the toolkit in hard copy print format included: noting how to record selections and rank by circling or highlighting the tick box, stars or Xs, and how to use the spaces to record details of selected BFs and to add BFs not listed should they arise. Participants reported referencing the instructions as they worked through the toolkit, and noted that while readily available on the print version, addition of a link to go back to the instructions in the computer-based formats would be of value.

### Meeting format

Although specific instructions for the meeting format were not provided, both sites elected to use a combination of small and large group discussions, with small groups composed of a heterogeneous mix of interested parties. In addition, both sites began the meeting with an introduction to the topic for discussion, including the evidence-based guideline itself, as well as local data highlighting the need for improved implementation of the selected guideline.

Participants from both sites were highly engaged and reported a number of benefits to the format utilised. They reported that the introduction to the topic and particularly the local data was helpful and needed to create a shared understanding of the need for improved implementation and set the stage for the discussions that followed.

Participants commented that the combination of small and large group discussions created an informal atmosphere. Small groups were noted to create a safe space for participants to share their perspectives, and large groups allowed for sharing of ideas and finalising selections through discussion and consensus. However, some noted that large groups can sometimes become too ‘enthusiastic’, which can be intimidating to some participants.

In addition, participants noted that even within the small groups, discussions could easily get off track. They recommended assigning a leader to help the group stay on topic or having the topic visible during discussions as a reminder. This suggestion was supported by the study teams’ observations, which noted groups in which an individual assumed a leadership role and kept the discussion on track were more efficient in working through the toolkit.

Participants at both sites commented on the value of including a mix of interested parties in each small group. They observed that the mixed groups allowed for better discussion and increased awareness and understanding of issues among different participant groups. In addition, mixed groups benefited from the opportunity for members to explain or clarify concepts within their areas of expertise, such as simplifying medical terminology for lay participants.

Participants also noted that the mixed groups allowed for varying perspectives to be brought forward, with some reporting that their understanding of issues was substantially improved as a result. The inclusion of participants from varying practice settings also allowed for solutions trialled and found successful at one or a limited number of sites to be shared broadly, with potential facilitators previously unknown to the majority of participants, brought forward as a result. Finally, participants noted that this increased understanding of others’ experiences and perspectives may help to improve cooperation and reduce blaming across participant groups.

Participants from both sites noted that while the mixed groups worked well for their setting and the range of participants represented, they agreed that mixed groups may not be ideal in some settings. An example provided noted the challenge of including key parties that may hold beliefs contradictory to the evidence (traditional healers, religious leaders). Participants suggested that in such cases the introduction would be particularly important to create awareness and understanding of the topic for discussion. In addition, participants recommended that in such cases, homogeneous small group discussions may be preferred to encourage participation, followed by presentations to and discussions in the large group to ensure all perspectives are considered.

#### Toolkit revisions

Based on the pilot testing, minor wording changes were made to the BFs listed, examples added to clarify two BFs and several additions made to the instructions to provide further guidance and suggestions for use. In addition, the Spanish and English print versions were updated and provided as attachments, and the print and audio of the worked example were updated and attached.

## Discussion

While KT approaches have been widely advocated to improve uptake of evidence into practice to improve programme planning, patient care and outcomes for a variety of health conditions in LMICs,^30^ lack of KT capacity^4^ and limited applicability of many existing KT tools to the unique challenges of LMIC health systems continue to limit their use.

We developed the maternal health implementation planning toolkit (https://maternal-health-implementation-planning-toolkit.ktss.ca) to address the need for a tool designed for use by non-experts and tailored to support implementation planning in LMICs. Our evaluation found the toolkit to be well received by participants who reported a number of benefits to content and organisation of both the toolkit and meeting format, with only minor changes to the instructions for use and wording of a few BFs listed suggested and incorporated to improve usability.

While a number of established tools may be used to assess BFs to implementation (eg, Theoretical Domains Framework, Consolidated Framework for Implementation Research, COM-B Behaviour Change Wheel), existing tools often require training for optimal use. Existing tools provide theory-informed frameworks of BFs that may be used to develop methods and tools (surveys, focus groups, etc) to assess BFs to implementation and guide analysis of findings. Guides to understanding the theoretical bases and framework categories are also provided. Although these tools allow for a comprehensive assessment of BFs, their approach requires training to ensure appropriate use and is often time-consuming, requiring several steps to arrive at an actionable understanding of BFs to inform implementation planning. To reduce the need for extensive training and facilitate use, while incorporating many key concepts from existing tools, our wording of BFs listed is designed to be straightforward, self-explanatory and comprehensive, while allowing space for the addition of novel BFs and elaboration of how BFs are of relevance to the context and evidence to be implemented to support the subsequent selection of implementation strategies.

In addition to the use of the toolkit to support initial implementation planning, the toolkit may also be of value during implementation to identify and guide response to BFs not initially identified or BFs arising as a result of changes to policy or contextual factors during implementation. Further study to evaluate the use of the toolkit for this purpose and to assess its potential to assess BFs post-implementation as a means to informing future implementation activities is needed.

A unique feature of the toolkit is the opportunity to rank the importance of BFs identified in order to highlight key issues and ensure challenges blocking implementation such as policy or legal issues are prioritised. In evaluating the toolkit, we found this option provided an additional opportunity for discussion and clarification and further informed consideration for selection of implementation strategies by emphasising BF targets likely to be most impactful.

Although focused on maternal health, our teams’ experience in conducting BF assessments across a range of health conditions (sepsis, tuberculosis) and settings (emergency medicine, public health) in LMICs suggests that the toolkit may be of value for implementation planning more broadly in LMICs and resource-limited health settings in high-income countries. Future work to assess and refine the tool for use in these settings is needed to extend its potential reach.

While the primary focus of the toolkit is on assessment of BFs to implementation, a basic guide to mapping BFs to evidence-based implementation strategies and additional resources to inform selection of implementation strategies is provided (CFIR-ERIC Implementation Strategy Matching Tool and SELECT tool), at the end of the toolkit. In evaluating the toolkit, we found that while many participants suggested or circled evidence-based implementation strategies listed in the toolkit, many added strategies that lacked evidence. Further evaluation to assess whether additional instructions to guide mapping of BFs is sufficient or whether an extension of the toolkit to provide guidance for selecting evidence-based implementation strategies may be of value.

### Strengths and limitations

There are several strengths to the process employed in developing and evaluating the toolkit. First, the toolkit was developed in collaboration with and in response to a need identified by our international partners for a simple toolkit for use by non-experts to support ongoing efforts to improve maternal health outcomes in LMICs, by improving uptake of maternal health evidence-based clinical guidelines, policies or health system interventions. Second, our pilot testing assessed use of the tool in substantially different contexts with respect to both organisation of maternal healthcare and the stage of maternal health (antenatal care, and labour and delivery) targeted for intervention. Finally, inclusion of a wide variety of end users in our pilot testing allowed for evaluation of the toolkit with individuals with a range of education, perspectives and experience, highlighting areas of strength and areas for improvement of the toolkit.

Our evaluation to date has several limitations. First, our pilot sites, while representing very different contexts, do not represent the range of contexts in which the toolkit may be applied and may therefore not have captured all areas for improvement. Future work to evaluate the toolkit in a wider range of countries and specific settings within countries is needed, to further refine the tool for use in a wider range of contexts. Second, as the evaluation was conducted in the setting of a funded project, implementers may find the cost of bringing together diverse representatives prohibitive. While strategies to conduct the assessment in alignment with other scheduled in-person activities or through online meetings may be of benefit in mitigating costs, these approaches require testing and may impose some restrictions on who can attend or reduce the highly valued interactive discussions relative to the in-person format. Third, as early phases of the project employed a theory-informed approach guided by the Theoretical Domains Framework (TDF) and Consolidate framework for implementation research (CFIR), we felt use of these frameworks in analysis of the toolkit testing data as well may be too prescriptive and elected to use a qualitative content approach. Alternative approaches to analysis may have yielded additional insights. Finally, as the evaluation of mapping of BFs and selection of implementation strategies was limited, and outcomes related to use of the toolkit in implementation planning were not evaluated, the effectiveness of the approach in improving both implementation and maternal health outcomes has not yet been established. Further study employing a mixed methods approach guided by an appropriate evaluation framework (eg, RE-AIM)(Reach Effectiveness Adoption Implementation Maintenance) is needed to examine if and how toolkit findings are incorporated into implementation strategies and to evaluate effectiveness of implementation planning guided by toolkit findings in improving implementation (such as acceptability, adoption and costs) and targeted maternal health (such as: increased knowledge and uptake of guideline recommendations) outcomes.

## Conclusion

The maternal health implementation planning toolkit provides a structured approach to assessment and ranking of BFs to implementation and a guide to support mapping of BFs to potential implementation strategies to address identified barriers and optimise facilitators. While further evaluation of the toolkit in the context of both maternal health and other health domains, across a wider range of LMICs and low resource contexts in high income countries, is needed, we believe the toolkit in its present form holds promise as a resource to support maternal health implementers in LMICs’ efforts to improve maternal health outcomes.

## Supplementary material

10.1136/bmjgh-2024-018616 online supplemental appendix 1

10.1136/bmjgh-2024-018616 online supplemental file 1

## Data Availability

Data are available upon reasonable request.
