# Supplementary material for: A knowledge translation toolkit for maternal health implementation planning in low- and middle-income countries: development and pilot evaluation in two countries
Source: BMJ Glob Health. 2025 Nov 29;10(11):e018616. doi: 10.1136/bmjgh-2024-018616 (PMC12666081; doi:10.1136/bmjgh-2024-018616)
Supplement: online supplemental appendix 1 [file bmjgh-10-11-s001.docx]

Appendix 1: Focus group Guide

Before we begin, I want to remind you of a few important points. The focus group is expected to last approximately 60 minutes and will be video recorded. Your participation is voluntary and you may refuse to answer any questions or withdraw from participation at any time without penalty. In addition, no personal information will be released to anyone outside the study team at any time, and your responses will be coded so that you cannot be identified.

Interview guide:

1. ***Do you have any questions?***

If yes, answer questions.

If no, may we proceed with the focus group now then?

1. ***First, I would like to ask you a few questions about yourself***.

Professional role/role in the meeting:

Clinician - specify (physician, nurse, midwife, other)

Academic/researcher -

Policy/decision maker -

Patient representative -

Have you had previous training or experience in implementation planning?

If yes, please briefly describe you past training/experience?

1. **Please tell me about your overall impression of implementation planning meeting?** *Possible Probes:*

-What did you think about the format of the meeting?

-What did you think worked well?

- How do you think the meeting format and/or process could have been improved?

1. **Please tell me about your overall impression of the toolkit?**

*Possible Probes:*

-What works well about the toolkit? What didn’t work well about the toolkit?

-What did you like about the design of the toolkit? What didn’t you like about the design of the toolkit?

-Do you think the toolkit would be helpful in implementation planning in your setting? Why or Why not?

- How likely are you to use the toolkit in planning future implementation efforts?

1. **What do you think about the format of the toolkit?**

*Possible Probes:*

- Were the toolkit instructions clear? (specifically probe for perspective from meeting leads and participants)

-Was the information in the toolkit presented clearly?

- How could the format of the toolkit be improved?

1. **What do you think about the content of the toolkit?**

- How could the content of the toolkit be improved?

- Are the Barriers and Facilitators listed relevant in your setting?

-Are there additional Barriers and Facilitators that you feel are missing or should be included in the toolkit?

- Is there anything not included in the toolkit that you think should be added?

1. **Do you have any other suggestions for how the toolkit can be improved?**
2. **Do you have any other suggestions on how future meetings like this one can be improved?**
3. **Do you have anything you want to add or clarify?**

Thank you for your time.
