# Supplementary material for: A knowledge translation toolkit for maternal health implementation planning in low- and middle-income countries: development and pilot evaluation in two countries
Source: BMJ Glob Health. 2025 Nov 29;10(11):e018616. doi: 10.1136/bmjgh-2024-018616 (PMC12666081; doi:10.1136/bmjgh-2024-018616)
Supplement: online supplemental file 1 [file bmjgh-10-11-s002.docx]

# **Reflexivity Statement**

1. **How does this study address local research and policy priorities?**

This study is part of a larger project to address a need identified by our international partners for a knowledge translation tool tailored for use by low- and middle-income country implementers. Improved implementation of maternal health evidence-based guidelines, practices and policies and research to support improved implementation, with the goal of improving maternal health outcomes is a priority for international organizations and local implementers in many low- and middle-income countries.

The implementation meeting topics in the pilot evaluation were selected by the local study team leads in collaboration with their networks to address a local priority for improved implementation in their context. The evaluation was designed to both test the toolkit and to provide data to support local implementers in developing plans to improve implementation of the selected topics.

1. **How were local researchers involved in study design?**

EA, MD, FA, and AG were involved in the development of the larger project, and part of the funding application, in support of this project. They along with MVO were involved in the design and development of the protocol for the qualitative study that informed development of the toolkit, MVO supervised the data collection and contributed to the analysis of the formative qualitative study, and all were included as authors on the manuscript. EA, MD, MVO, FA, AG and MA, contributed to the design and development of the current study, design and translation of study documents as needed, led selection of the topic for and led the implementation planning meetings as part of the pilot evaluation. All contributed to the writing of the local meeting reports and current manuscript, and led dissemination of meeting findings within their local networks.

1. **How has funding been used to support the local research team?**

Funding for this project was provided to local study teams to support study activities, including: meeting costs and salary support to local team leads and stipends for students providing logistical support to the project.

1. **How are research staff who conducted data collection acknowledged?**

Data collection for the current study was conducted by the Toronto study team (LPR, KG).

1. **Do all members of the research partnership have access to study data?**

Data from the preliminary work is held both a local sites and Toronto site. Summary data from the implementation planning meetings has been provided to the local teams.

1. **How was data used to develop analytical skills within the partnership?**

MVO participated in analysis of data from the preliminary qualitative study, where she developed experience with an approach to qualitative analysis utilizing knowledge translation frameworks. Analysis of the toolkit evaluation data was conducted by the Toronto study team. In leading the implementation planning meetings, local teams developed experience in conduct and analysis of BF assessments and selection of implementation strategies to guide implementation planning.

1. **How have research partners collaborated in interpreting study data?**

Local teams collaborated in the interpretation of the data, through discussion of meeting

proceedings in real time providing context to ensure understanding of the data provided by

participants, and provided feedback and clarification to improve interpretation of local findings

for preparation of the meeting reports and interpretation of combined data to inform toolkit

revisions and preparation of the current manuscript.

1. **How were research partners supported to develop writing skills?**

The study team is largely composed of experienced researchers, with extensive

research and technical report publications. While this article did not substantially

support development of writing skills among the junior study authors, opportunities for

first author publication of local data are available and will be supported by senior

authors.

1. **How will research products be shared to address local needs?**

A meeting report outlining findings of the implementation planning meeting and

recommendations for next steps, has been provided to local team leads to share with

meeting participants and with their wider networks. In addition, open access

publications from the project will be made available to partners and participants.

1. **How is the leadership, contribution and ownership of this work by LMIC researchers recognised within the authorship?**

All study team members in both the current and preliminary study are included as co-authors on

the study manuscripts. In addition, local study team members are co-authors on the meeting

reports. While more than half the co-authors are LMIC researchers, we acknowledge that the

first author position is based in a HIC. The primary reason for this is first authors involvement in

all phases of the larger project, including formative work leading to development of the project,

and focus of the current manuscript on evaluation of the toolkit itself rather than on findings

of the implementation planning meetings proceedings, provided through meeting reports to

local teams.

1. **How have early career researchers across the partnership been included within the authorship team?**

MVO is an early career researcher based in Argentina. She has contributed to the writing

and has been included as a co-author on all manuscripts and the Argentina meeting

report.

1. **How has gender balance been addressed within the authorship?**

Three authors are male (AG, EA, FA) and seven authors female (LPR, KG, MVO, MD, MA, CF, SS)

1. **How has the project contributed to training of LMIC researchers?**

The majority of study team members are experienced researchers. A graduate

student in Ghana gained experience in research conduct, by providing logistical support

to the study and participating in the meeting and focus group discussions.

1. **How has the project contributed to improvements in local infrastructure?**

This project has not directly contributed to improvements in local infrastructure.

1. **What safeguarding procedures were used to protect local study participants and researchers?**

Ethical approval was obtained by the local ethics boards for all participating sites. Specific

attention to creating a safe space for participants to share perspectives during the meeting and

focus group. In addition, opportunities provided for participants to share their concerns with the

study made available in real time during breaks in the meeting and through provision of contact

information for both the local and international study team members, in the consent forms with

copies provided to participants.

Local study teams led/co-led by senior researchers with extensive experience collaborating on

international projects.
